# Perinatal depression and mental health uptake referral rate in an obstetric service

**DOI:** 10.1038/s41598-023-33832-6

**Published:** 2023-07-07

**Authors:** Francisca Tato Fernandes, Ana Beatriz de Almeida, Mónica Fernandes, Rosa Correia, Rui Magalhães, Graça Buchner, Jorge Braga, Paula Freitas

**Affiliations:** 1 Instituto de Ciências Biomédicas Abel Salazar, Universidade do Porto, Porto, Portugal; 2 Gynaecology and Obstetrics Department, Cento Materno Infantil do Norte, Centro Hospitalar Universitário do Porto, Porto, Portugal; 3 Clinical Psychology, Cento Materno Infantil do Norte, Centro Hospitalar Universitário do Porto, Porto, Portugal; 4 Psychiatric Department, Cento Materno Infantil do Norte, Centro Hospitalar Universitário do Porto, Porto, Portugal; 5 CINTESIS, Instituto de Ciências Biomédicas Abel Salazar, Universidade do Porto, Porto, Portugal

**Keywords:** Depression, Health services

## Abstract

Perinatal depression is an important indicator of mothers’ mental health. Studies have been carried out to identify and characterize women at risk of such affective disorder. The aim of this study is to assess mothers’ adherence to our perinatal depression screening and eventual follow-up by a multidisciplinary team, including mental health and obstetrics professionals. Ultimately, a risk profile for the uptake rate of referral was described to psychological support. Pregnant women from a maternity of a tertiary center with on-site assessment and treatment (n = 2163) were included in this study. The identification of women at risk for depression was based on a two-question screening and the EPDS scale. Demographic and obstetric data were obtained from medical records. The number of screening evaluations, the uptake referral rate and the compliance to treatment were analyzed. Logistic regression was used to predict a risk profile for adherence. Among 2163 enrolled in the protocol, 10.2% screened positive for depression. Of these, 51.8% accepted referral for mental health assistance. 74.9% were compliant to Psychology appointments and 74.1% to Psychiatry appointments. Women who had a previous history of depression were more likely to accept referral for mental health support. With this study, we were able to understand the behaviour of this population towards the screening protocol we offer. Women with a previous history of depression are more likely to accept mental health assistance.

## Introduction

Pregnancy is known to be a period of vulnerability in which emotions between astonishment and insecurity may sprout, and it is a challenging period for both physical and mental women’s health. Perinatal depression is by far the most common mood disorder, affecting one in every seven women^1^. It is defined by the presence of depressive symptoms that occur during pregnancy or until one year after delivery^2^. Evaluating perinatal depression can be challenging and efforts have been made to bring up professionals dedicated to maternal health disorders, able to identify perinatal mood and anxiety disorders.

Evidence suggests that only 20% of depressed pregnant women receive adequate treatment^3^. As we know, untreated depression can result in poor adherence to healthcare, exacerbation of previous physical conditions, substance abuse, suicide and other adverse outcomes^1,3^. Therefore, it becomes imperative to standardize a screening protocol for perinatal depression, as well as an appropriate follow-up for these women^1,4^.

If detection does not foresee treatment, its value is limited^5^. Moreover, identifying barriers can be a way to overcome a lack of compliance. Barriers can be present on different levels. Kim et al.^6^ studied barriers that could affect the linkage of women to a screening protocol. On a patient level, women said that lack of time was the biggest barrier. They also mentioned spontaneous improvement of symptoms and that they used another support. By evaluating the provider’s interaction with the patient, women have also reported a lack of empathy and unavailability of the provider to schedule an appointment in case of need of the patient. Currently, the cost of the appointment and the insurance not being able to cover for the expenses were reported as a common barrier.

Risk factors that contribute to the development of perinatal depression have been identified: personal or familial psychiatric history, adverse life events, lack of support and others^3^. Moreover, 20% of pregnant women in developed countries have a chronic physical condition, which may be exacerbated during pregnancy, resulting in a higher vulnerability of developing a mental disorder. Evidence suggests that obstetric complications as hypertensive disorders, gestational diabetes, pregnancy loss and premature birth might have a positive relationship with depression^7–11^.

Most of the above-mentioned complications can be closely assessed by obstetricians during appointments. Therefore, they have a unique opportunity to, not only follow-up physical illnesses, but also to early identify changes in women's mental health^12^. Initially, talking about these issues could be seen as a barrier, however Byatt et al.^13^ showed that pregnant women not only appreciate, but wish to actively discuss mental health with their obstetrician.

All mental health services should evaluate the engagement level of their patients, since the rate of non-compliance to scheduled appointments is 20%, two-times greater than in other clinical areas^14^. Also, there is no universal definition for treatment drop-out or withdrawal of treatment, which difficult analysis^15^. Considering perinatal mental health, research suggests that approximately half of positive screened gravidae for depression and/or anxiety attend a mental health specialist^6^. Causes for this should be explored, as lack of literacy, since 30% of women reported no information about depression^16^.

In conclusion, the aim of this study is to assess therapeutic adherence to a proposed protocol carried out by a local maternity considering sociodemographic and obstetric factors. Ultimately, the study will focus on describing women who uptake referral for psychological assistance.

## Methods

A cross-sectional study involving a retrospective record review was carried out in a six-month period, between 1st July 2019 and 31th December 2019.

The Centro Hospitalar da Universidade do Porto/Instituto de Ciências Biomédicas Abel Salazar (CHUP/ICBAS) Ethics Committee approved the study as well as waivered this experiment of Informed Consent since it was not necessary to collect data beyond those that were already registered. Pregnant women were aware of the registration of the data that was used during consultations. No woman was submitted to any other interview or procedure. It is a retrospective study without intervention. Ultimately, the data to be processed was anonymized and not likely to jeopardize the rights and freedoms of their holders. All methods were carried out in accordance with relevant guidelines and regulations.

### Protocol description and tools

The Obstetric Department, supported by the Mental Health Team (MHT), at *Centro Materno-Infantil do Norte Albino Aroso* (CMIN), a local maternity of a tertiary hospital—*Centro Hospitalar Universitário do Porto* (CHUP)–, has implemented a protocol that aims to detect depression in pregnant and postpartum women. This MHT is a group of psychologists and psychiatrists who work in the same unit, having close and direct contact with the Obstetric Department.

The procedure foresees the fulfilling of a Screening Questionnaire (SQ), in three distinct moments, which correspond to obstetrical visits; namely: the first contact with the Obstetric Department (SQ 1); the last month of pregnancy appointment (SQ 2) and the postpartum review assessment (SQ 3). The SQ is applied during the nursing assessment of each appointment. Subsequently, the questionnaires were analyzed by the MHT.

For the screening itself, we used the Edinburgh Postpartum Depression Scale (EPDS), which was validated by Cox et al.^17^ for antenatal and postnatal periods. This screening tool consists of ten straightforward questions, easily and quickly answered by women. Cox et al. affirms satisfactory results for a cut-off of 12/13, once sensitivity and specificity showed up to be 78% and 86%, respectively.

The SQ is composed of two screen questions plus the 10-question EPDS. Therefore, considering them as two steps:

Step 1: two screen questions with “Yes/No” answers; *1—During the last month, did you feel frequently unmotivated, depressed or hopeless?; 2—During the last month, did you feel frequently disinterest or dissatisfaction in carrying out activities?*

If at least one answer is “Yes”, women proceed to Step 2, which is EPDS. In other words, the completion of the EPDS (Step 2) is dependent on the previous answers.

The screening evaluation is considered positive if the women completed Step 2 and the EPDS score is equal or higher than 12. A negative result corresponds to two “No” answers in Step 1 or, in Step 2, an EPDS score lower than 12.

Finally, women have to answer the following, also with “Yes/No” answers: *1-Do you already have psychological support? 2—Do you feel in need of psychological support?* If the SQ result is positive and the woman wants support, the MHT makes a referral and schedules a Psychology appointment. If the woman does not want the referral to be made, it is considered a refusal.

### Sample and data collection

All women who attended our facility during the time of the study were included at first. Those whose SQ registration was correct and enabled statistical analysis we eligible. Sociodemographic, obstetric data and past medical history were collected retrospectively through medical records, as well as the data concerning the referral to psychological assistance and its respective uptake and compliance. This was the information that could be collected since the program that health professionals use during appointments does not include questioning women about race, education and income status.

This screening program has an on-site assessment and treatment, with an established referral network to mental health professionals.

**Figure 1:**
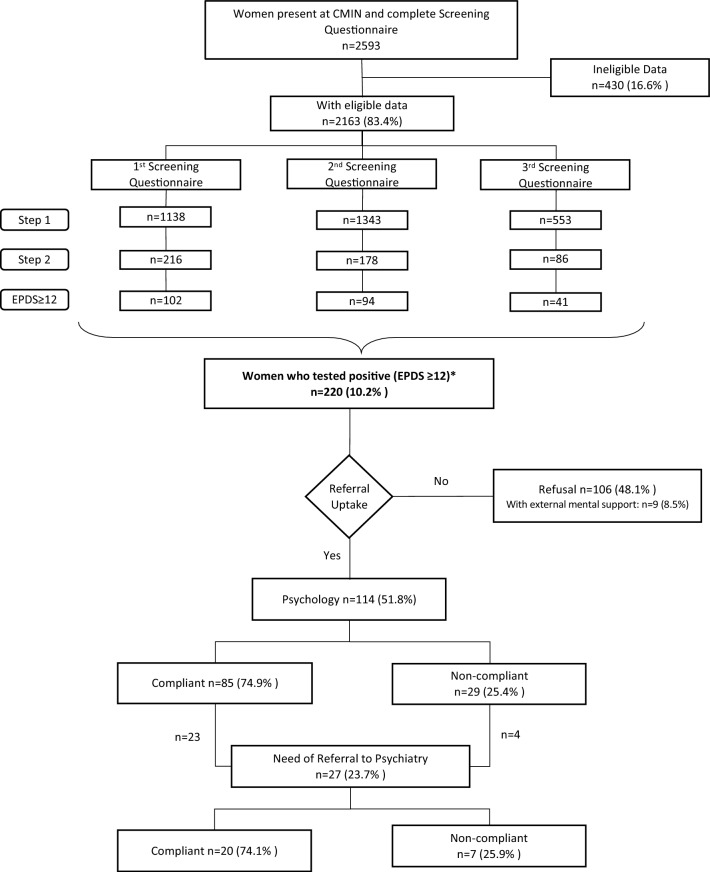
Descriptive flowchart illustrating our perinatal depression screening protocol. *Women can have more than one positive screening questionnaire.

**Figure 2:**
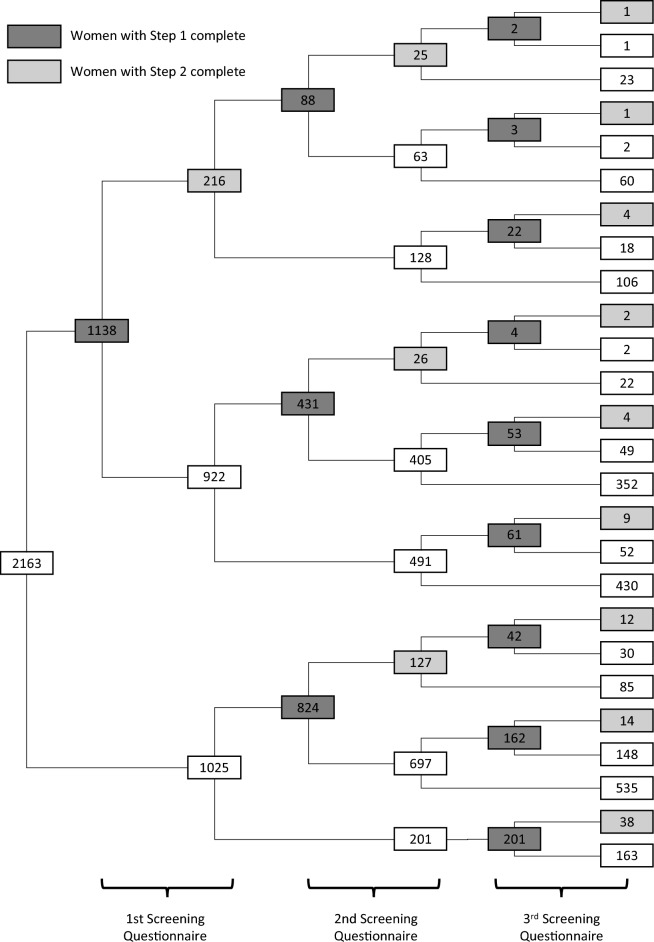
Dendrogram with number of participants in each screening moment.

Figures 1 and 2 describe schematically the process.

### Measures

After the fulfilment of the SQ, the referral acceptance and refusal were registered. The uptake referral rate (number of women who accepted referral among those to whom it was offered) was obtained through clinical records as well as the compliance to Psychology and Psychiatry appointments. Compliance designates women who attended at least one appointment, unlike non-compliance that refers to those cases that had full non-attendance.

The variables analyzed were divided into three moments considering the timeline of a pregnancy and postpartum. Within the preconception variables, “Previous Condition”, assembles mental and/or physical maternal comorbidity; “Psychiatric History”, contemplates proper diagnoses with prescribed medication and/or assertion of the woman as having a regular psychological or psychiatric external accompaniment; “Maternal Comorbidity” gathers those who have a physical condition. Also, data were collected for the number of pregnancies, children, poor pregnancy outcomes and the type of conception. “Pathology” during the antenatal period concerns medical circumstances that only occurred during the index pregnancy. The same applies to “Hospitalization” and the presence of “Fetal Malformation”.

### Statistical analysis

Sample description was performed using counts and percentages for categorical variables, and the mean and standard deviation for continuous variables. Women who screened positive in EPDS test were compared with the rest. Among women who tested positive, a comparison was made between those who agreed or not to be referred for a psychology appointment. Women's ages were compared using an independent sample t-test. Categorical variables were compared using a chi-square test or a Fisher exact test when appropriate. A binary logistic regression model was used to identify independent predictors of referral for Psychological appointments among positive screened women. A p-value of 0.05 was considered the limit for wrongly rejecting the null hypothesis. Data analysis was performed using IBM SPSS Statistics version 27.

## Results

### Primary analysis

Among 2593 pregnant and postpartum women who were enrolled in our Perinatal Depression Screening Protocol between July and December 2019, 427 women were excluded due to irregularities in data registration.

Out of the 2163 women analyzed, 220 had a positive screening (10.2%). Of them, 114 accepted psychological referrals (51.8%) and 27 were forwarded to psychiatric clinical assessment. Evaluating refusals, only 9.5% corresponded to women with ongoing external accompaniment by a mental health professional.

Compliance for both Psychology and Psychiatry appointments was similar among women (about 75%). The median attendance of compliant pregnant women for Psychology appointments was 75% (IQR: 50–90%), which means that 25% of women were present in less than 50% of scheduled appointments. Concerning Psychiatry appointments, similar results were observed (median of 75%, IQR: 50–100%).

Figure 1 describes schematically each step of the protocol and it briefly shows the number of SQs completed. No differences were found between referral uptake rates concerning the three screening moments (*p* = 0.249). However, among those who accepted the referral, the difference showed a trend: those with a positive SQ 1 or 2 will more likely be compliant to appointments (*p* = 0.066).

Figure 2 outlines the number of SQs that were performed in each moment and demonstrates possible path women could have made, since they could have completed SQ 1 and/or SQ 2 and/or SQ 3.

### Secondary analysis

Overall, 220 women (10.2%) screened positive for depression. The mean maternal age was 32.1 years. These women were more likely to be multigravidae, to have at least a child and to have a previous pregnancy loss (induced abortion and/or a miscarriage and/or an ectopic pregnancy). A psychiatric background (depression, anxiety and/or other psychopathology) was also frequent. The remaining variables did not show significant differences (Table 1).

**Table 1:** Description of mothers' past obstetric history, comorbidities and delivery characteristics.

	EPDS screen score				*P* value	Uptake of referral				*P* value
	< 12		> 12			Yes		No		
Characteristics	n	%	n	%		n	%	n	%	
No. of women	1943		220			114		106		
Mean age (SD)	31.5	(5.6)	32.1	(6.0)	0.142	32.3	(6.0)	31.6	(5.7)	0.582
Preconception										
Any comorbidity	625	32.2	131	59.5	< 0.001	77	67.5	54	50.9	0.012
Psychiatric history^a^	54	2.8	55	25.0	< 0.001	42	36.8	13	12.3	< 0.001
Depression	33	1.7	35	15.9	< 0.001	30	26.3	5	4.7	< 0.001
Anxiety	17	0.9	9	4.1	< 0.001	8	7.0	1	0.9	0.023
Other psychopathology	7	0.4	6	2.7	< 0.001	5	4.4	1	0.9	0.117
Maternal physical comorbidity	571	29.4	76	34.5	0.113	35	30.7	41	38.7	0.214
Multigravida	1043	53.7	149	67.7	< 0.001	78	68.4	71	67.0	0.819
Number of children					< 0.001					0.600
No children	1146	59.0	101	45.9		56	49.1	45	42.5	
One child	566	29.1	79	35.9		38	33.3	41	38.7	
More than one child	231	11.9	40	18.2		20	17.5	20	18.9	
Previous pregnancy loss	489	25.2	73	33.2	0.010	45	39.5	28	26.4	0.040
Pharmacologic abortion	93	4.8	18	8.2	0.031	11	9.6	7	6.6	0.410
Medical interruption	43	2.2	8	3.6	0.189	6	5.3	2	1.9	0.181
Miscarriage/ectopic pregnancy	385	19.8	56	25.5	0.050	34	29.8	22	20.8	0.123
Assisted procreative technology	72	3.7	5	2.3	0.277	1	0.9	4	3.8	0.150
During pregnancy										
Pregnancy pathology^b^	598	33.0	67	32.4	0.854	38	35.2	29	29.3	0.365
Hospitalization^c^	174	9.7	23	11.2	0.502	15	13.9	8	8.2	0.193
Geminal pregnancy^d^	19	1.0	2	0.9	0.910	1	0.9	1	1	0.952
Fetal malformation^e^	64	3.6	7	3.4	0.896	5	4.6	2	2.0	0.306
Delivery	1636		197			104		93		
Delivery type										
Eutocic	821	50.2	95	48.2	0.598	50	48.1	45	48.4	0.965
Dystocic	815	49.8	102	51.8		54	51.9	48	51.6	
Vacuum delivery	332	20.3	35	17.8	0.584	16	15.4	19	20.4	0.710
Forceps delivery	39	2.4	6	3.0		4	3.8	2	2.2	
Cesarian section	444	27.1	61	31.0		34	32.7	27	29.0	
Neonatal death	14	0.9	4	2.0	0.114	3	2.9	1	1.1	0.369
Premature	149	9.1	18	9.1	0.989	13	12.5	5	5.4	0.083
Prolonged hospitalization										
Mother	368	22.5	51	25.9	0.284	30	28.8	21	22.6	0.316
Newborn	181	11.1	16	8.1	0.208	9	8.7	7	7.5	0.773

^a^Women may have simultaneous conditions; ^b^144 missing information; ^c^162 missing information; ^d^88 missing information; ^e^167 missing information.

This group of at-risk women for perinatal depression was analyzed under the same conditions, enabling the comparison between those who accepted referral (n = 114) and those who did not (n = 106). Differences were only seen in preconception characteristics: a psychiatric background and a previous pregnancy loss were more frequent in women who accepted referrals. For the remaining variables, no differences were observed (Table 1).

The results of the univariable analysis showed that mothers with a previous history of depression were more likely to uptake the referral, as well as those with a previous pregnancy loss. Prematurity showed a trend of a higher probability of referral acceptance (OR = 2.51, 0.86–7.35), however, it didn’t reach statistical significance (*p* = 0.092).

After the multivariable analysis, history of previous depression was the only variable with statistical significance (OR = 6.11, 2.08–17.93; *p* < 0.001). Although not significant, having already an infant, showed a trend (*p* = 0.093). The remaining characteristics were not predictive of up-taking referral, as shown in Table 2.

**Table 2:** Univariable and multivariable models for uptake referral characterization (n = 197*).

	Univariate			Multivariate		
Characteristics	OR	95% CI	*P* value	OR	95% CI	*P* value
Age	1.01	0.97–1.05	0.686	1.03	0.97–1.09	0.339
Preconception						
Depression	7.21	2.68–19.4	< 0.001	6.11	2.08–17.9	< 0.001
Maternal physical comorbidity	0.70	0.40–1.23	0.215	0.95	0.50–1.90	0.946
Multigravida	0.94	0.53–1.65	0.819	1.96	0.56–6.89	0.295
Children	0.76	0.45–1.30	0.322	0.38	0.13–1.17	0.093
Previous pregnancy loss	1.82	1.03–3.22	0.041	1.08	0.46–2.56	0.855
Assisted procreative technology	0.23	0.02–2.05	0.186	0.18	0.01–2.45	0.200
During pregnancy						
Pregnancy pathology	1.31	0.73–2.35	0.366	0.76	0.34–1.70	0.499
Hospitalization	1.82	0.73–4.49	0.197	1.52	0.48–4.81	0.475
Fetal malformation	2.33	0.44–12.3	0.319	2.88	0.45–18.4	0.262
Delivery						
Eutocic	1.00		(0.788)	1.00		(0.612)
Instrument delivery	0.86	0.41–1.78	0.680	0.66	0.29–1.53	0.334
Cesarian section	1.13	0.59–2.16	0.704	0.98	0.45–2.17	0.968
Neonatal death	2.73	0.28–26.7	0.388	1.28	0.08–21.1	0.861
Premature	2.51	0.86–7.35	0.092	3.00	0.75–12.1	0.122
Prolonged hospitalization						
Mother	1.39	0.73–2.65	0.317	1.03	0.44–2.40	0.949
Newborn	1.16	0.42–3.26	0.773	0.95	0.28–3.16	0.928

*Excluded 23 cases with missing information.

## Discussion

This study focused on managing women at risk for depression during the perinatal period. The prevalence of depression screened in our study (10.2%) was similar to other published data, suggesting our protocol should be a recognized procedure. However, when detection does not foresee treatment, its value is limited.

As studies suggest, an on-site assessment enables a better mothers’ follow-up, with a higher uptake rate for mental health referral^18,19^. It also improves engagement and decreases stigma^20^, since their standard care occurs in the same environment. Xue’s et al.^21^ findings also affirm that an on-site assessment or referral has a higher rate of treatment than a referral to a mental health service (60% vs 32%).

Our referral uptake rate was 51.8%, suggesting that more than half of the positive screened women showed a willingness to have psychological support. To our knowledge, only three other studies had a higher referral uptake rate: 65%^22^, 100%^23^ and 74.1%^24^. Despite this, only Segre et al. referred women according to their will. We believe this higher rate may be explained by the fact that screening was done at the women’s home, with the possibility to explain deeply depression and its possible effects. Venkatesh and Kallem et al. did not consider the women’s will.

By evaluating compliance, only Chen et al.^25^ had a greater rate than our findings and we believe it could be related to having a smaller population size. We believe our compliance results are satisfying, which may be due to an on-site assessment with a functional referral network, but also based on considering the mothers’ will of undergoing mental support. In our study, the referral refusal rate was of 48.1% which is an alarming fact, since only 10% of these women with a positive screening had external accompaniment. Scholle and Kelleher^26^ found that many women do not want professional advice on depression and prefer to rely on informal sources or family/friends. This may be an explanation for our high rate of refusals, since these were women who affirmed not feeling the need of psychological support.

Upon our results concerning the risk profile, it can definitely be affirmed that women with a history of depression adhere on a larger scale to psychological support. Similar results were seen in other studies^24^. Depression disorders have a high probability of recurrence, and since pregnancy is a period of susceptibility, it can be considered as a trigger. Based on these facts and our results, we suggest the addition of a new screen question in Step 1: “*Have you had a previous depression disorder?”*. If so, women should be required to complete the EPDS. This way, we would have a higher probability of detecting women at risk for perinatal depression and, possibly, a higher compliance to mental health support.

This approach has already been implemented in Southwest Michigan^27^, and it has been discovered that women who have previously had depression are more likely to experience postpartum depression. This strengthens our suggestion to include a new screen question in the Screening Questionnaire, not only in postpartum but also during pregnancy, according to our findings. Furthermore, the findings of this study refer that an early onset of postpartum depression predict a longer period of depression as well as a higher severity.

Although our study did not aim to identify barriers or reasons for refusals, some should be discussed. Previous research suggests that the main reasons for non-attendance to treatment are lack of time, difficulty in finding childcare, the belief of being capable of using their own resources, not being informed or even asked about mental health^5,14^. Stigma is still poorly understood, with dichotomous opinions. Furthermore, studies suggest the implementation of a protocol combining a SQ, education and motivational support could engage those non-compliant women into treatment^5,14,28^.

As strengths of this study, it should be mentioned, firstly, the fact that we designed a protocol, which can be an asset to the scientific community, eventually it can be implemented in other health care settings. Secondly, the fact that we have a multidisciplinary team working in the same direction and that there is a specialized appointment for this condition in our own maternity hospital. Finally, we believe that our sample was of a considerable size, as we only analyzed women enrolled during a six-month period.

There are several limitations in our study. Firstly, a considerable ineligible data that was excluded. Secondly, the reasons for non-compliance were not recorded, which would be a great value of information to exponentiate the efficiency of the protocol. Thirdly, we did not consider the accurate period of mental disorder episodes (one or several years ago).

## Conclusion

Our study emphasizes the importance of an on-site assessment. Having mental healthcare services integrated with obstetric and pediatric-related services increases women’s engagement.

According to our results, women with a previous history of depression were more likely to accept mental health assistance. Therefore, we consider that it is of utmost importance to include in screening questionnaires women’s previous psychiatric history, namely, if they have had a depression disorder at some point in life. This may contribute to a precocious detection of perinatal depression, diminishing its prevalence and improving its treatment.

## Data Availability

The datasets used and/or analysed during the current study available from the corresponding author on reasonable request.

## Abbreviations

DSM-V: Diagnostic and statistical manual of mental disorders V
ICD-10: International classification of diseases
EPDS: Edinburgh postnatal depression scale
MHT: Mental health team
CMIN: Centro materno-infantil do Norte Albino Aroso
CHUP: Centro Hospitalar Universitário do Porto
SQ: Screening questionnaire
